# TDR Inversion for Water Localization and Uncertainty Evaluation

**DOI:** 10.3390/s26082432

**Published:** 2026-04-15

**Authors:** Marco Scarpetta, Maurizio Spadavecchia, Francesco Adamo, Gregorio Andria, Nicola Giaquinto

**Affiliations:** Department of Electrical and Information Engineering, Polytechnic University of Bari, Via E. Orabona 4, 70125 Bari, Italy; marco.scarpetta@poliba.it (M.S.); francesco.adamo@poliba.it (F.A.); gregorio.andria@poliba.it (G.A.); nicola.giaquinto@poliba.it (N.G.)

**Keywords:** TDR inversion, distributed sensing, water localization, uncertainty evaluation, gray-box modeling, cyber-physical measurement system

## Abstract

This work presents the application of a Time-Domain Reflectometry (TDR) inversion algorithm for localizing water along a bi-wire cable acting as a distributed sensing element (SE), and for evaluating the uncertainty of the water position measurement. The TDR inversion relies on a simplified yet effective gray-box circuital model of the measurement system that, without attempting a full-wave electromagnetic (EM) simulation, reproduces with good accuracy any actually observed reflectograms. The model parameters are estimated from a single acquired reflectogram so as to reproduce the measured signal, without a prior EM characterization of the system components. The model provides the water localization and enables extensive simulation campaigns under realistic variations in water position, stimulus pulse duration, and disturbance effects. A specific measurement setup, designed to perform repeated measurements in controlled laboratory conditions, is analyzed in detail as a case study. The water localization error of the measurement system is statistically evaluated in terms of confidence intervals, bias, and standard deviation, by means of simulated measurements of the model, with different water positions and TDR pulse durations. Then, the uncertainty evaluation is validated through 45 actual measurements, using multiple SEs, and the same water positions and pulse durations. The work proves the viability and the performance of the presented TDR inversion method for both localization measurements and for their uncertainty evaluation under different experimental conditions. More generally, it establishes a general framework for TDR measurements and uncertainty evaluation combining physical modeling, simulation-based uncertainty evaluation, and experimental verification.

## 1. Introduction

Time-Domain Reflectometry (TDR) was originally developed as a diagnostic technique for locating simple faults in transmission lines, such as open or short circuits [1,2]. The method relies on the analysis of reflections generated by impedance discontinuities along a cable when a stimulus signal is injected at one end. By measuring the time delay between the transmitted and reflected signals, the position of a discontinuity can be accurately determined. With the advancement of the technique, the same principle has been extended to the detection of soft faults, i.e., regions where the electrical properties of a cable are altered without producing a complete interruption or short circuit. Identifying soft faults can be performed through expert visual interpretation of reflectograms, but the growing complexity of modern systems has motivated the development of rigorous analysis methods based on the mathematical modeling of the reflection process [3,4,5].

The natural evolution of this concept led to the development of distributed sensing based on TDR. In this case, a cable or transmission line is not merely a signal path but acts as a sensing element (SE) that responds to variations in the surrounding medium. A classic example is the use of buried cables for monitoring soil movements in landslide-prone slopes, where mechanical stress produces localized variations in impedance along the line [6,7,8]. More generally, distributed TDR sensing relies on detecting and localizing variations in the effective dielectric constant around the SE, which directly affects the local capacitance and propagation velocity of the signal.

TDR-based distributed sensing has found application in several domains, including leak detection in buried water pipes [9,10], monitoring of concrete curing and drying processes [11,12], and geotechnical or structural health monitoring [13,14,15,16]. Most of these applications are ultimately related to the localization of regions containing water, whose high dielectric constant significantly modifies the electromagnetic properties of the surrounding medium. In leak detection, for instance, water escaping from a pipe infiltrates the nearby soil, causing a local increase in dielectric constant and generating a reflection that can be detected in the time-domain response.

The analysis of a reflectogram for the localization of water, or more generally for the identification of regions exhibiting significant variations in dielectric constant, can be performed through several approaches. The simplest techniques are based on the manual identification of features in the reflectogram, followed by straightforward calculations that relate their temporal positions to spatial coordinates and their amplitudes to the magnitude of the underlying variation [6,7,9,13]. More advanced methods, on the other hand, make use of signal processing and machine learning algorithms, including neural networks trained to automatically recognize and interpret characteristic patterns in the measured reflectograms [17,18].

A more ambitious and general approach is known as TDR inversion or spatial TDR. In this case, the objective is to reconstruct the spatial profile of the dielectric constant that generated the observed reflectogram. In principle, an exact solution of this inverse problem requires detailed prior knowledge of the SE, the connection cables, and the instrumentation, as well as the measurement of multiple reflectograms under different conditions. Consequently, rigorous inversion is feasible only in simplified or idealized configurations [19,20,21].

A practical approach to TDR inversion and water leak localization is provided by a recently developed method based on a simplified yet accurate gray-box circuit model of the measurement setup [10]. The model, including the distributed dielectric constant profile along the SE, can be identified directly from a single TDR measurement, without requiring a detailed electromagnetic characterization of the system components. Although approximate, the model is capable of faithfully reproducing the reflectograms observed in real experimental setups, which are often irregular and affected by various nonidealities.

In this work, the previously developed TDR inversion framework [10] is refined and extended to enhance the accuracy of water localization. In particular, the inversion is organized into two steps: the first provides a flexible coarse reconstruction of the distributed dielectric profile along the SE, while the second refines this estimate using a compact parametric representation of the water region.

The second main contribution provided by this paper is the framework for uncertainty evaluation of TDR-based localization. The proposed approach exploits the model of the complete measurement system, identified from experimental data, to accurately reproduce reflectograms observed in real conditions. The fact that the entire measurement chain, including instrumentation, connection cables, sensing element, and the disturbance conditions observed in a given setup, can be effectively modeled on the basis of a single representative measurement enables the generation of a large number of realistic simulated reflectograms under controlled parameter variations. This makes it possible to perform extensive Monte Carlo analyses and to statistically quantify localization errors, thereby providing a rigorous and practically applicable assessment of measurement uncertainty. In realistic field scenarios, such an uncertainty evaluation would not be feasible through repeated controlled experiments, which makes the proposed model-based approach particularly suitable for quantifying performance.

The refined inversion method and the proposed uncertainty evaluation framework are both applied and assessed on a specific case study presented in this paper. The uncertainty of the localization results is quantified through several hundred realistically modeled simulations, each generating reflectograms corresponding to hypothetical experimental scenarios and incorporating perturbations representative of real-world disturbances. The simulation-based uncertainty evaluation is then experimentally validated through 45 independent measurements, showing close agreement between predicted and observed localization errors. Although the study refers to a specific measurement setup, the methodology is general and can be readily applied to other TDR-based localization systems and methodologies.

Accordingly, the main novelties of this work with respect to [10] can be summarized as follows: (i) the introduction of a two-step inversion strategy for improved localization accuracy; (ii) the development of a simulation-based framework for uncertainty evaluation; and (iii) the experimental validation of the proposed uncertainty assessment.

The paper is organized as follows. Section 2 describes the TDR-based water localization method, including the experimental setup and the refined inversion algorithm. Section 3 presents the uncertainty evaluation methodology, which combines simulated and experimental analyses. Section 4 reports and discusses the results of the uncertainty analysis. Section 5 concludes the paper and summarizes the main findings.

## 2. TDR Inversion for Water Localization

### 2.1. TDR-Based Water Localization: An Example

The typical field setup for TDR-based water localization in buried water pipes comprises a distributed SE installed near the pipe. The SE consists of an unshielded bi-wire cable, whose effective dielectric constant depends on the surrounding medium, and is strongly increased by the presence of water. A TDR unit is connected to one end of the SE through a shielded connection cable, such as a coaxial line, which passes through an inspection well. The TDR unit transmits a stimulus signal and records the reflections along the SE. When a leak is present, water infiltrating the soil locally increases the effective dielectric constant, altering the signal propagation and producing a detectable reflection. By analyzing these reflections, the leak can be localized, although the achievable accuracy depends on the analysis method and the clarity of the reflection.

**Figure 1:**
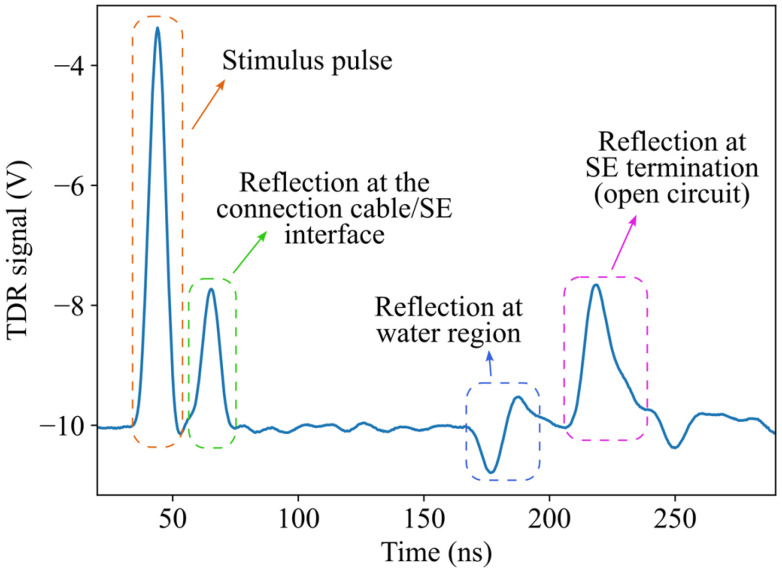
Example of the voltage signal measured by the TDR unit in the presence of a water region. The stimulus pulse and major reflections are annotated. In this example, the reflection at the water region is clearly visible (which is not always the case in practice), while secondary reflections and oscillations illustrate the non-ideal response of the system under field conditions.

An example of a TDR signal measured using the setup previously described is shown in Figure 1, with the main features annotated. The stimulus signal, which in this case is a pulse-like waveform, appears first. This is followed by a reflection at the interface between the connection cable and the SE. The reflection associated with the water region is then observed and exhibits the characteristic s-shaped pattern typical of a capacitive fault, which results from a localized increase in dielectric constant. The reflection from the open end of the SE follows, along with multiple secondary reflections. The non-ideal behavior of the SE and its surrounding environment is evident in the oscillatory disturbances visible even before the reflection caused by the water region.

Even in this relatively favorable example, localizing the water region is not straightforward, and it is much less clear how to assign an objective, non-arbitrary uncertainty to the localization. Traditional approaches rely on measuring the time of flight of the reflections corresponding to the SE ends and the water region, then converting them into spatial coordinates through a linear transformation. However, the reflections are clearly distorted by the system’s linear dynamic response, making the identification of their exact timing ambiguous and, consequently, the associated uncertainty poorly defined. While the problem of reflection timing has been extensively addressed in the literature [22], it is not the focus of this work; instead, we adopt a TDR inversion approach, aimed at reconstructing the dielectric profile most consistent with the measured signal, thereby providing a more consistent framework for uncertainty evaluation.

### 2.2. TDR Measurement System Modeling

**Figure 2:**
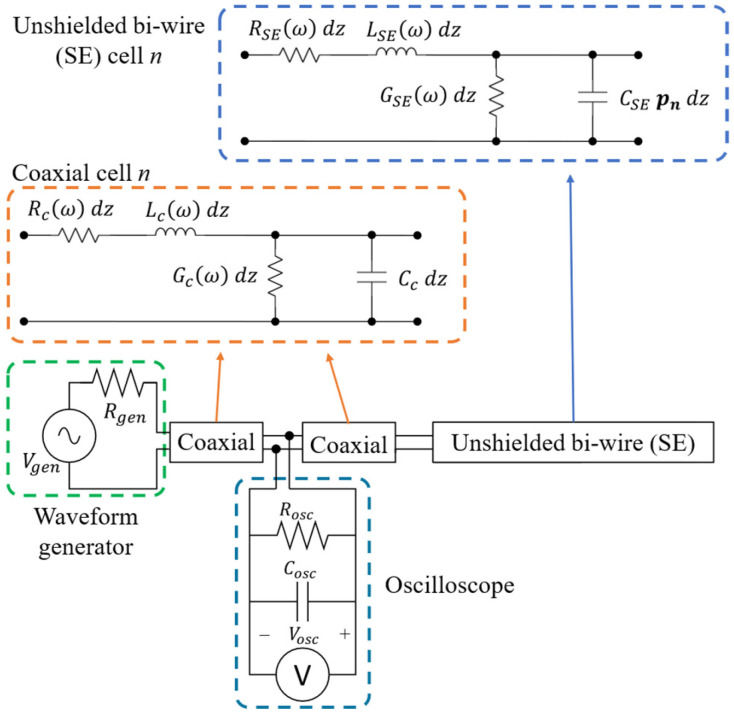
Model of the measurement system, including the instruments (waveform generator, oscilloscope), the coaxial connection cables (RG58) and the unshielded bi-wire sensing element (SE).

The model, shown in Figure 2, is a distributed-parameter circuit representation of the measurement system. Both the coaxial connection cables and the unshielded bi-wire SE are modeled as transmission lines (TLs), discretized into elementary cells with identical structure, as described in [10,23]. A full-wave electromagnetic simulation would, in principle, be more rigorous, but it would also result in a model that is unfeasible to identify and impractical to use.

The frequency-dependent behavior of the per-unit-length primary parameters of each cell is modeled as follows:

(1) $$R\left(f\right)=R_{0}+R_{1}\sqrt{f},$$

(2) $$L\left(f\right)=L_{0}+L_{1}/\sqrt{1+f/f_{0}},$$

(3) $$G\left(f\right)=G_{0}f,$$

(4) $$C=C_{0},$$

where f denotes frequency. These expressions account for skin effect and dielectric losses in coaxial and twin-lead cables, providing an engineering-level approximation that is valid in the frequency range of interest (up to about 200 MHz), as shown in [17]. More accurate models of transmission-line parameters are available in the literature (e.g., [24] for coaxial cables), but they typically require a detailed electromagnetic characterization of the system and are not suitable for direct identification from reflectometric measurements.

Although simplified and not intended to capture effects such as non-TEM propagation, radiation, or external interference, the model reproduces with high fidelity the reflectograms observed in experimental setups, while remaining mathematically manageable. This makes it suitable for parameter identification and for estimating localized variations in the dielectric constant along the sensing element. The efficiency of the model also makes it practical for generating the large number of reflectograms required in applications such as random-search optimization.

The model must also reproduce an assigned dielectric constant profile along the SE. To this end, each cell at position z is associated with a multiplicative coefficient p(z) that scales its capacitance. This allows the model to represent localized perturbations of dielectric properties, such as those caused by water regions, as well as nuisance factors including material inhomogeneities or environmental variations. In the inversion process, this distributed profile is the primary unknown to be estimated, alongside the global model parameters (e.g., $R_{0},\;R_{1},\;L_{0},L_{1},\;f_{0},G_{0},C_{0}$, with distinct values for the coaxial cables and the SE).

The circuital model in Figure 2 is simulated using the LineLab transmission line simulator [23], which computes reflectograms in the frequency domain and transforms them into the time domain. This approach enables accurate modeling of frequency-dependent effects, making the model suitable for TDR analysis under field conditions.

### 2.3. TDR Inversion

The TDR inversion introduced in [10] consists of adjusting the parameters of the system model in Figure 2 so that the simulated reflectogram, obtained from the known input pulse, matches as closely as possible the measured one. In this framework, the inversion problem is reduced to an optimization task over the model parameters.

Not all parameters are optimized. Practical experience has demonstrated that for the internal resistance and capacitance of the instruments, the nominal values given in the specifications are sufficiently accurate, and so are the lengths of the connection cables obtained by simple geometric measurements. Consequently, they are kept fixed. Likewise, the length of the sensing element $l_{SE}$ is considered known; leaving it free would cause parameter degeneracy with L and C of the SE, which control the propagation velocity.

Accordingly, the optimization focuses on:

- Set (a): primary parameters of the coaxial cables $R_{0,c},R_{1,c},L_{0,c},L_{1,c},f_{0,c},G_{0,c},C_{0,c}$;
- Set (b): primary parameters of the sensing element $R_{0,SE},R_{1,SE},L_{0,SE},L_{1,SE},f_{0,SE},G_{0,SE},C_{0,SE}$;
- Set (c): parameters defining the capacitance profile p(z).

The random search algorithm used for optimization introduces successive small random perturbations to a suitable initial guess, seeking the parameter sets that align the simulated signal with the measured one, under the least-squares criterion. At each iteration, only a randomly selected subset of the fitted parameters is perturbed. The number of perturbed parameters is set to 20% of the total fitted parameters. The perturbations are drawn from zero-mean distributions with standard deviations defined for each parameter, set to 5% of the corresponding initial value. During the optimization, these amplitudes are updated adaptively: whenever an improved solution is found, the perturbation standard deviation of each parameter is recomputed from the accepted perturbations for that parameter. In addition, if no improvement is obtained for a prescribed number of iterations, the perturbation amplitudes are progressively reduced, so as to favor local refinement of the solution. The optimization process stops when the mean squared error between the measured and simulated signals decreases by less than 0.1% over the last 500 iterations. The nonconvex nature of the optimization problem makes the choice of the initial guess very important in order to avoid local minima and solutions with no physical meaning.

The optimization follows a two-step approach. In both steps, all three parameter sets (a), (b) and (c) are estimated, but using different models for the capacitance profile p(z).

The initial guess for sets (a) and (b) is obtained by fitting the model to a reflectogram acquired by the measurement system in the absence of water. This operation already yields a good estimation of the primary parameters of both the coaxial cables and the SE, taking into account their actual physical condition (soil surrounding the SE, and other relevant factors).

**Figure 3:**
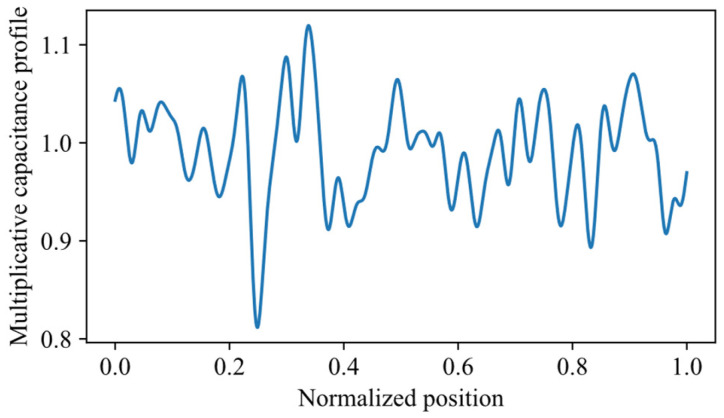
Example of a dielectric profile $p\left(z\right)$ generated by summing $N_{G}=100$ Gaussian pulses with variable (positive or negative) amplitude $A_{G,i}$, plotted as a function of the normalized position $z/l_{SE}$. Such a profile is used as the first-step estimate of the capacitance distribution in the TDR inversion process.

The first optimization step is based on estimating the profile $p\left(z\right)$ as a train of $N_{G}=100$ Gaussian functions (see example in Figure 3). The corresponding mathematical expression, which uses only $N_{G}$ real parameters $A_{G,i}$, positive or negative, is:

(5) $$p\left(z\right)=p_{G}\left(z\right)=1+\sum_{i=0}^{N_{G}-1}A_{G,i}\exp\left(-\frac{{\left(z-\frac{l_{SE}}{N_{G}-1}i\right)}^{2}}{2{\left(\frac{l_{SE}}{N_{G}-1}\right)}^{2}}\right).$$

The initial guess for the parameters $A_{G,i}$ is zero, which corresponds to $p\left(z\right)=p_{G}(z)\equiv 1$ (and a flat capacitance profile equal to $C_{0}$). The optimization algorithm then iteratively updates these parameters, together with sets (a) and (b), to better fit the measured reflectogram. The perturbation standard deviation for each Gaussian coefficient is set to a constant value (0.05), enabling the progressive introduction of localized variations along the sensing element. This functional model of p(z) is particularly suitable when no prior information is available about the dielectric profile, because the algorithm can progressively introduce localized variations at $N_{G}$ equispaced positions along the SE, allowing for a sufficiently flexible and adaptive reconstruction of the dielectric constant distribution. On the other hand, the fixed length and position of the Gaussian pulses inherently limit the spatial resolution of the estimation. Therefore, this kind of profile, with its fixed and relatively small set of parameters, is a trade-off between achieving a reliable first localization of the water region position and maintaining an optimization process that is both fast and computationally efficient.

In the second optimization step, the dielectric profile is represented with a simpler rectangular model:

(6) $$p_{R}\left(z\right)=1+A_{R}\cdot rect\left(\frac{z-z_{c}}{w}\right),$$

where $A_{R},\;z_{c}$ and w denote the amplitude, center, and width of the rectangle, respectively.

Compared to the Gaussian expansion, this three-parameter model is less suitable for reproducing the measured reflectogram in detail; however, since the position $z_{c}$ is an explicit free parameter, it is more effective in accurately localizing the water region, provided that a good initial guess for $z_{c}$ is available. The initial values of $A_{R}$, $z_{c}$, and w are obtained by least-squares fitting of the rectangular function (6) to the capacitance profile estimated in the first optimization step, whereas the initial values of parameter sets (a) and (b) are those resulting from the first step. Starting from these values, the optimization is repeated with the rectangular model, refining the three parameters, together with sets (a) and (b), to obtain an accurate and computationally efficient estimation of the leak location and extent.

This two-step optimization procedure represents a flexible framework that can be adapted or simplified depending on specific needs (e.g., using an alternative profile model in the first or second optimization stage). In any case, the subsequent uncertainty evaluation procedure remains valid, since it relies on the model of the system and its use for replicating experiments under a wide range of hypothetical conditions. Unlike a conventional validation based on a limited number of practical tests, this approach enables a much more robust assessment of the method’s performance.

## 3. TDR Inversion for Uncertainty Evaluation

The uncertainty evaluation is obtained with simulated measurements, and is validated by actual measurements on a physical laboratory setup. Since the uncertainty evaluation requires the realization of a model of the measurement system, we present first the laboratory setup and the related experimental tests. The parameters of the system model used for simulations, depicted in Figure 2, must in fact be determined from prior knowledge of the experimental setup and from a limited set of actual measurements.

### 3.1. Laboratory Setup

The laboratory setup was designed to meet the specific requirements of the study, aimed at validating the uncertainty evaluation using the results of many repeated experiments. The requirements are:

- Enabling water regions to be introduced at different positions;
- Allowing repeated introduction of a water region at the same position;
- Allowing multiple replicas of the same setup to be constructed (five in this study);
- Ensuring that the entire system remains manageable within a laboratory environment.

**Figure 4:**
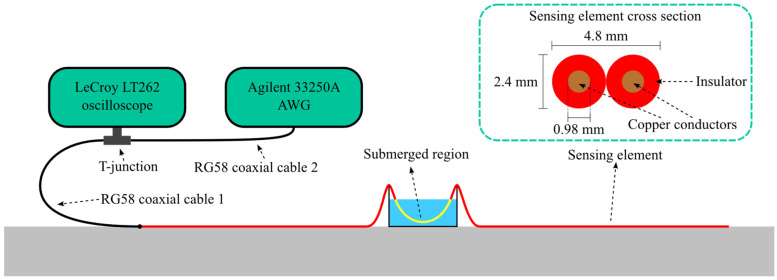
Laboratory experimental setup used to replicate the water leak localization measurement scenario. The system includes an arbitrary waveform generator (Agilent 33250A AWG), an oscilloscope (LeCroy LT262), and a sensing element where leaks can be introduced at controlled positions.

The setup is shown in Figure 4. The SE is placed on the ground and terminated with an open circuit. Water leaks are emulated by submerging a segment of the SE in a container filled with water. The TDR unit consists of an 33250A (Agilent, Santa Clara, CA, USA) arbitrary waveform generator (AWG) for stimulus signal generation, and a LT262 (LeCroy, Chestnut Ridge, NY, USA) oscilloscope for signal acquisition. Two RG58 coaxial cables, 1 m and 2 m long respectively, connect the AWG to the oscilloscope, and the oscilloscope to the SE, which is 15 m long. The Agilent 33250A is capable of generating Gaussian-shaped pulses with a minimum standard deviation of 3.1 ns. The LeCroy LT262 oscilloscope provides an analog input bandwidth of 350 MHz and has a specified input impedance of 1 MΩ in parallel with 16 pF.

In this setup, the SE is laid on the ground—a circumstance that does not introduce simplifications in the TDR inversion procedure. It simplifies greatly the construction of five identical physical replicas of the setup, and the collection of a large and reliable dataset of independent measurements in repeatability conditions, needed for validating the simulation-based uncertainty evaluation. Analyzing a setup with a SE buried in soil is of course of interest, but is not proposed in the present study, since it does not add anything substantial to the uncertainty evaluation methodology. Choosing such a setup just makes its realization in five identical and controlled conditions much more expensive, and the acquisition of many repeated measurements, which requires wetting and drying the soils multiple times in the same places to reproduce the same humidity conditions, more difficult and time-consuming.

### 3.2. Experimental Tests

**Figure 5:**
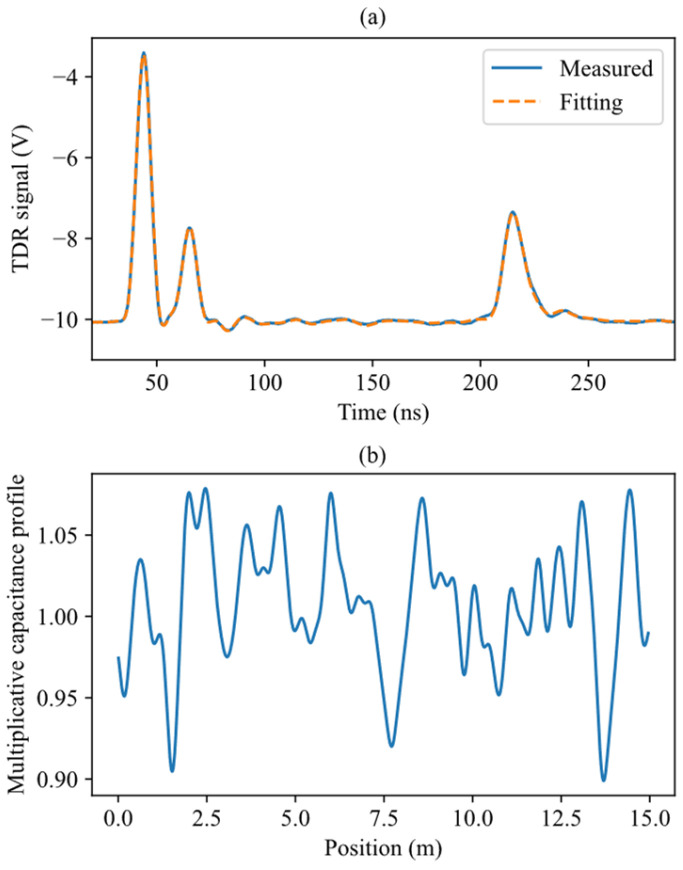
Measurement of the laboratory setup of Figure 4 in dry conditions. (**a**) Measured reflectogram in the time domain. (**b**) Capacitance profile resulting from the TDR inversion. The oscillation in the capacitance profile reproduces that observable in the reflectogram.

The first experimental test consists of taking a reflectogram in dry condition, followed by the first step of the TDR inversion method. The result is shown in Figure 5 and in Table 1. The procedure gives the primary parameters of the coaxial cables and of the SE in dry conditions (Table 1), which serve as initial guesses for the random search of TDR inversion, and also to build the system model (reproduce the measurement setup in simulated measurements). The measured capacitance profile is also needed for the model, which must be subject to disturbances similar to those observed in the actual setup.

It should be noted that the parameters reported in Table 1 are identified as part of the gray-box model of the measurement system and are not intended to represent independently measured physical quantities. Their role is to enable an accurate reproduction of the measured reflectograms and to define the baseline model used in the subsequent simulation-based analysis.

The other experimental tests aim at collecting measurements to validate the uncertainty evaluation. These tests are performed using five SEs, made of the same kind of bi-wire and with the same nominal length of 15 m; the water regions are realized by submerging a 60 cm segment of the SE in water.

The effect of the water position on measurement uncertainty is investigated by varying the center of the region $z_{c}$ within the sensing element. Three locations are considered: the center of the sensing element at 7.5 m, and two positions closer to the terminations, located at 3.5 m and 11.5 m. This analysis aims to determine how the distance of the leak from the beginning of the sensing element influences the accuracy of its localization.

**Figure 6:**
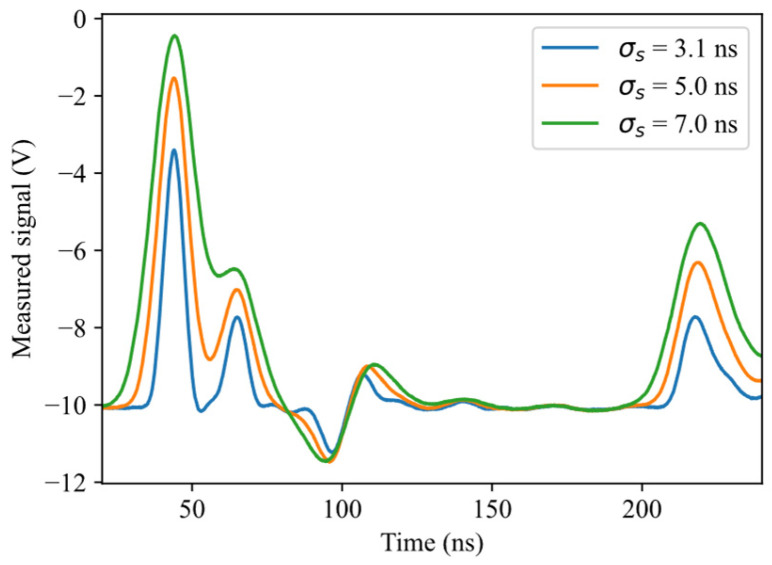
Measured signals for three different transmitted pulse widths. The plot shows the main portion of the signal, from the transmitted pulse to the reflection at the SE termination.

The influence of the stimulus signal duration on water localization accuracy is also examined. The reason is that narrower pulses allow a better time resolution, but are also more strongly attenuated along the SE, reducing the signal-to-noise ratio. In each experiment, the stimulus signal is approximately a Gaussian pulse, with standard deviation selected from three possible values: 3.1 ns (the minimum allowed by the Agilent 33250A arbitrary waveform generator), 5 ns, and 7 ns. As shown in Figure 6, a standard deviation of 7 ns causes great overlap between reflections, and has therefore been considered as a practical maximum for the considered experimental setup.

The nominal parameters of the experimental tests with water are summarized in Table 2 for clarity.

The total number of experimental tests is equal to (no. of water locations) × (no. of pulse widths) × (no. of physical SEs) = 3 × 3 × 5 = 45. The 45 separate measurements provide a solid statistical basis for assessing the uncertainty of the measurement method, but obtaining such a dataset is possible only in a laboratory setup of the kind shown in Figure 4, not in a real on-field scenario.

In the following, we assess the measurement uncertainty using just simulated experiments, as if we had a real-world measurement setup, where producing a number of measurements with exactly known water regions is not feasible. The core of the simulation-based method is of course the model in Figure 2, whose parameters are adjusted to accurately reproduce the behavior of the actual measurement setup. The experimental measurements will be used to verify the uncertainty assessment provided by the simulated measurements.

### 3.3. Simulated Tests

The basis of the simulated tests is the model in Figure 2, with parameters suitable to reproduce actual laboratory measurements.

The primary parameters of the simulated cables are set at the values in Table 1, that is, those of the laboratory setup measured under dry conditions. Simulated cable lengths, water region positions, water region width, and the standard deviations of the Gaussian pulses, are the same as the experimental tests, listed in Table 2.

**Figure 7:**
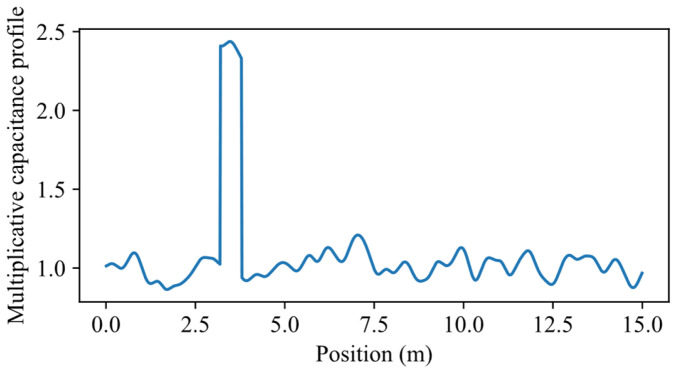
Example of multiplicative capacitive profile employed in simulations. The water leak here is centered at $z_{c}$ = 3.5 m.

Water regions are simulated by multiplicative capacitance profiles p(z) of the kind depicted in Figure 7. Such profiles are the sum of the rectangular function of Equation (6) and an oscillating floor similar to that actually measured in the experimental setup, shown in Figure 5. This oscillatory component represents the combined effect of small-scale inhomogeneities and imperfections of the sensing element and its surrounding medium, including mechanical irregularities (e.g., cable deformations and torsions), variations in cable geometry, and non-uniformity of the surrounding medium (e.g., soil heterogeneity). The rectangular function of Equation (6) has the position and width as given in Table 2, and amplitude randomly drawn from a uniform distribution in the range from 1.75 to 2.75. These limits were selected based on amplitudes obtained from the fitting of the experimental measurements in the presence of water described in Section 3.2, which lay approximately within the interval [1.9, 2.6]. The range was slightly extended to account for variability arising from nuisance effects, such as model simplifications and environmental fluctuations affecting the effective dielectric properties of the sensing element. The Gaussian functions of Equation (5) have amplitude values $A_{G,i}$ drawn from a normal distribution, having a zero mean and standard deviation of 0.05. The chosen parameters were derived from the analysis of experimental measurements under dry conditions, such as that shown in Figure 5, where the optimized amplitudes $A_{G,i}$ were well approximated by a normal distribution with zero mean and a standard deviation of 0.03. Overall, the random amplitude of the rectangle, and the random Gaussian floor produce, for identical water leaks, reflectograms with a variability similar to that observable in practice.

Each combination of stimulus duration and leak position was tested in 100 simulated measurements. In each repetition, a different random seed was used to generate the oscillating component of the capacitance profile to assess the impact of external disturbances on the estimation results. The TDR inversion method described in Section 2.3 was then applied to each simulated reflectogram to estimate the corresponding capacitance profile. To further simulate a realistic scenario, in which the primary parameters of the coaxial cables and the sensing element are not accurately known, the initial guess of these parameters was randomly perturbed before starting the optimization. The perturbation was drawn from a normal distribution with zero mean and a standard deviation equal to 5% of the absolute value of each parameter.

Once the rectangular profile was estimated, the error in water leak localization was computed as the difference between the estimated center parameter $z_{c}$ and the true position used to generate the simulation. To ensure a consistent and fair comparison among all combinations of stimulus durations and leak positions, the same 100 random seeds were used to generate the oscillating floor and the initial parameter guesses across tests for all the combinations of stimulus duration and leak position.

## 4. Results and Discussion

In this Section we first present in some detail the obtained simulated reflectograms and simulated measurements, and the consequent uncertainty assessment. Afterwards, we compare this assessment with the results of actual measurements.

### 4.1. Simulation Results and Uncertainty Assessment

**Figure 8:**
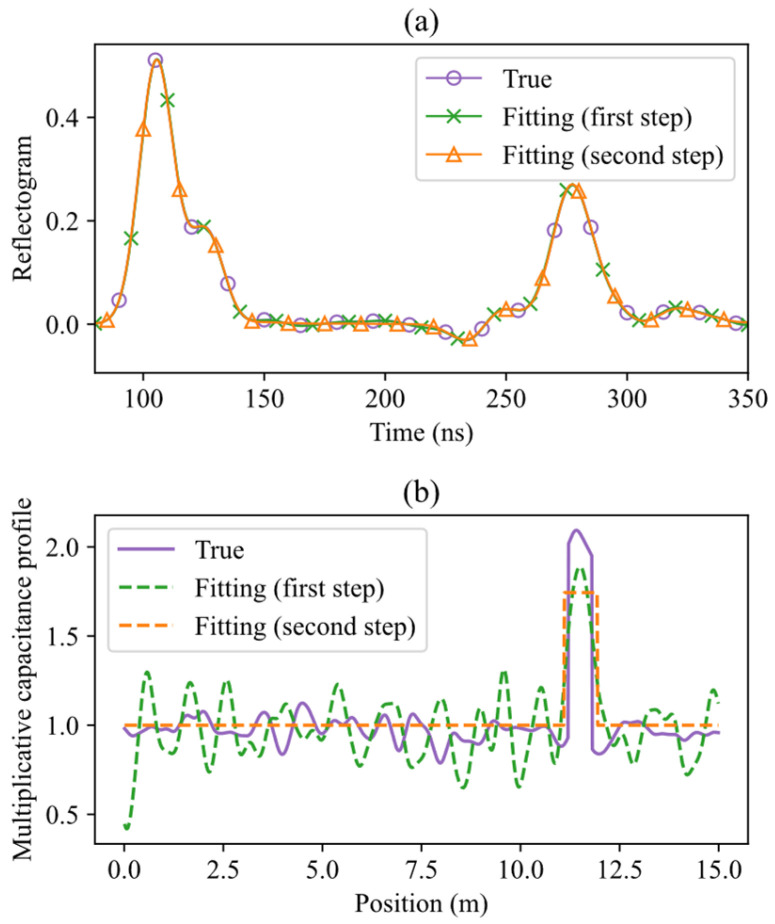
Water leak localization using simulated TDR data for a leak centered at $z_{c}$ = 11.5 m, with $\sigma_{s}$ = 7 ns. This is a challenging case, where the leak-induced reflection is not clearly distinguishable from other oscillations in the reflectogram. (**a**) True (simulated) reflectogram, and fitted reflectograms after the first and second optimization steps. (**b**) True capacitance profile used in the simulation, and estimated capacitance profiles after each step.

**Figure 9:**
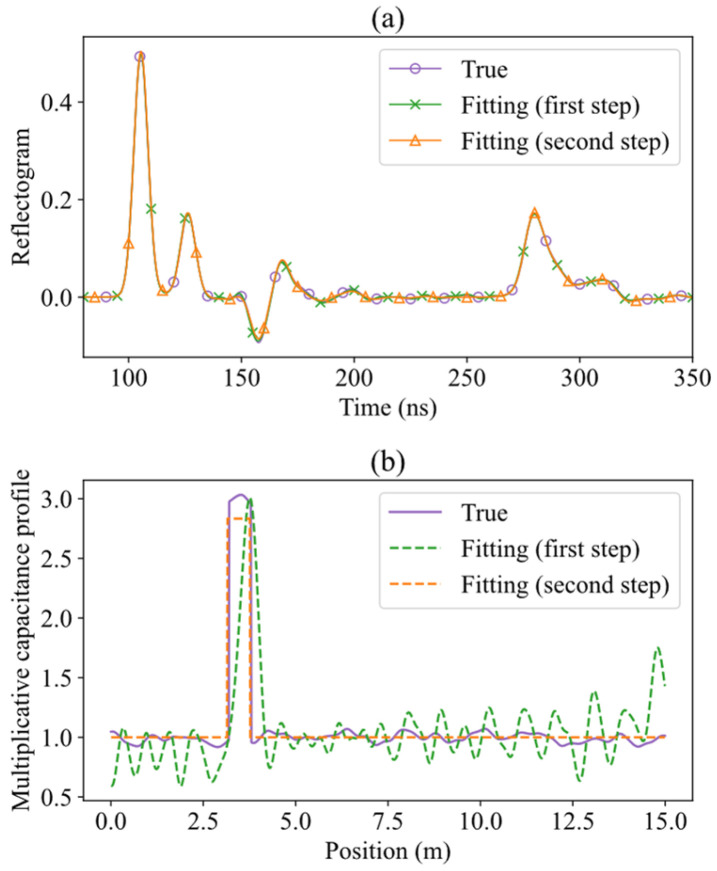
Water leak localization using simulated TDR data for a leak centered at $z_{c}$ = 3.5 m, with $\sigma_{s}$ = 3.1 ns. Here, the leak-induced reflection is clearly separated from other oscillations in the reflectogram. (**a**) True (simulated) reflectogram, and fitted reflectograms after the first and second optimization steps. (**b**) True capacitance profile used in the simulation, and estimated capacitance profiles after each step.

Two examples of reflectograms “measured” in simulated measurements, and of results obtained applying the TDR inversion method, are shown in Figure 8 and Figure 9.

The first example ($\sigma_{s}=7\;ns$, $z_{c}=11.5\;m$) corresponds to a challenging case. In the simulated reflectogram of Figure 8a, the leak-induced reflection is not clearly distinguishable, and the use of traditional feature-based TDR analysis methods may lead to quite different measurements and poor localization accuracy. The estimated capacitance profiles after the first and second optimization steps of the TDR inversion are shown in Figure 8b. The leak is already visible after the first optimization step, even if with oscillations due to the introduced nuisance factors, and the inherently limited resolution of the inversion at this stage. Starting from this estimate, the second optimization step produces a good reconstruction of the true rectangular profile, and an accurate localization of the leak. This example highlights the robustness and effectiveness of the illustrated TDR inversion method, even when applied to signals with low visual interpretability.

In contrast, the second example ($\sigma_{s}=3.1\;ns$, $z_{c}=3.5\;m$) shown in Figure 9 depicts a scenario where the leak-induced reflection is stronger and more distinct. The difference, with respect to the previous case, is that the estimated rectangular profile is really very close to the simulated one. The leak is, of course, accurately localized.

**Figure 10:**
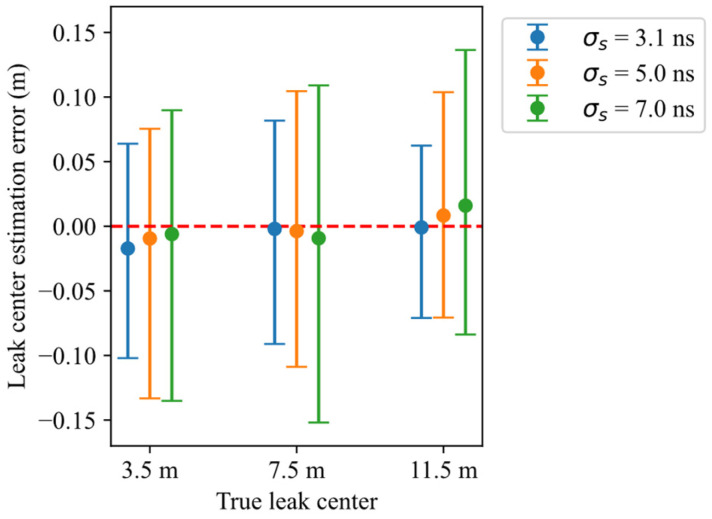
Errors in estimating water leak centers $z_{c}$ in the simulations, represented as intervals encompassing 95% of the error values. The lower whisker, circle and upper whisker correspond to the 2.5th, 50th (median), and 97.5th percentiles, respectively.

The statistical analysis of the 900 simulated measurements (100 repetitions for each of nine setup conditions reported in Table 2) is presented in Figure 10, which reports the median, 2.5th percentile, and 97.5th percentile of the water leak localization errors, defined as the difference between the estimated and true leak center $z_{c}$. The error distributions are quite similar for different leak locations and pulse widths, with a very small nonzero median, and a slightly lower dispersion for shorter pulse durations. For the shortest pulse duration (*σ_s_* = 3.1 ns), the interval containing 95% of the localization errors is [−10.2 cm, 6.4 cm], while across all simulated cases the corresponding interval is [−11.6 cm, 9.9 cm]. Overall, the results depict a localization accuracy that is little affected by leak location and pulse duration.

Statistics in terms of mean and standard deviation of the localization errors are summarized in Table 3. For each leak position, the lowest mean and standard deviation values are highlighted in bold. These results are in line with the trend observed in Figure 10, with the most accurate estimations generally associated with shorter stimulus pulse durations. The only exception is observed for $\sigma_{s}=7\;ns$ and $z_{c}=3.5\;m$, where the mean error is slightly lower than that obtained with $\sigma_{s}=3.1\;ns$ at the same leak position. Overall, the standard deviation of the estimates is between 3.9 and 6.1 cm, and the bias is between −1.65 and 1.45 cm. The worst performances, not surprisingly, are associated with the more distant leaks and the larger impulse widths; they are not, however, much worse than the best performance.

### 4.2. Experimental Measurements and Validation of Uncertainty

**Figure 11:**
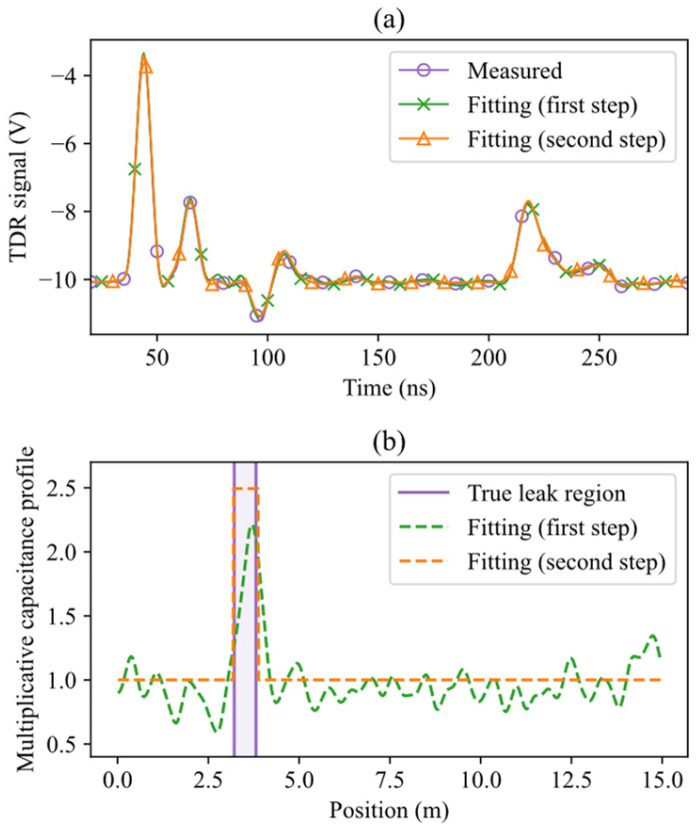
Water leak localization using experimental TDR data for a leak centered at $z_{c}$ = 3.5 m, with $\sigma_{s}$ = 3.1 ns. (**a**) Measured reflectogram, and fitted reflectograms after the first and second optimization steps. (**b**) Actual water leak region, and estimated capacitance profiles after each step.

An example of a reflectogram obtained with actual laboratory measurements and water leak localization via TDR inversion is shown in Figure 11, which is the experimental counterpart of the simulated case presented in Figure 9. This result has been obtained with a leak positioned at $z_{c}$ = 3.5 m and a stimulus pulse with a standard deviation $\sigma_{s}$ = 3.1 ns. As shown in Figure 11a, the leak-induced reflection is clearly visible in the measured reflectogram, which is expected for a “near” leak observed with a “short” pulse. The capacitance profiles estimated at the first stage of the optimization process, displayed in Figure 11b, clearly show the presence of the leak, and give its position with good approximation. The rectangle estimated at the second stage closely matches the actual location of the water region. The example illustrates the method’s reliable and accurate work when applied to experimental data.

**Figure 12:**
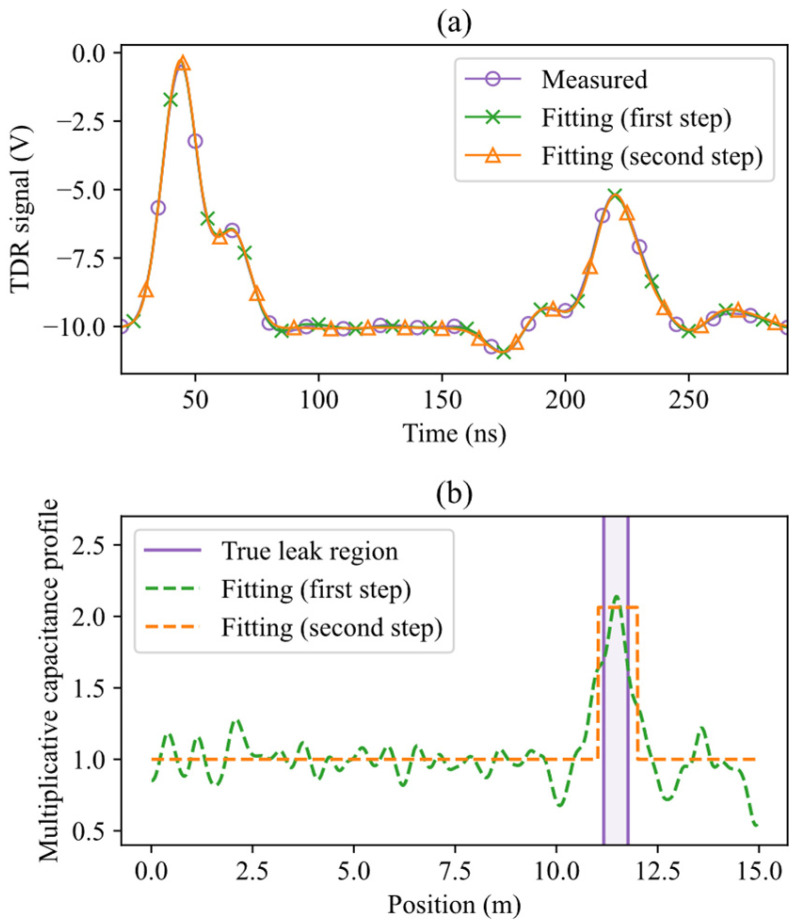
Water leak localization using experimental TDR data for a leak centered at $z_{c}$ = 11.5 m, with $\sigma_{s}$ = 7 ns. (**a**) Measured reflectogram, and fitted reflectograms after the first and second optimization steps. (**b**) Actual water leak region, and estimated capacitance profiles after each step.

An example obtained under more challenging conditions, with a stimulus pulse possessing a standard deviation of $\sigma_{s}$ = 7 ns and a water region centered at 11.5 m, is shown in Figure 12. In this case the reflection associated with the water region is weak and partially masked by oscillatory disturbances and secondary reflections, making visual interpretation of the reflectogram difficult. Nevertheless, the proposed TDR inversion method successfully identifies and localizes the water region, reconstructing a capacitance profile that closely matches the true distribution.

**Figure 13:**
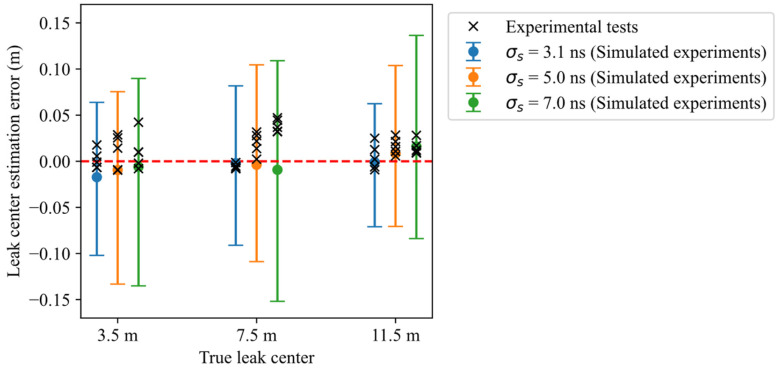
Errors in estimating water leak centers $z_{c}$ in the experiments, compared with simulation results. The error values obtained in the experiments are all within the corresponding intervals (2.5th to 97.5th percentiles) computed with simulations.

To evaluate the consistency between experimental measurement errors and simulation-based uncertainty evaluation, the errors from all 45 experimental measurements are plotted in Figure 13, alongside the corresponding simulation results already shown in Figure 10. Each experimental error is plotted over the intervals representing the 2.5th and 97.5th percentiles of the simulated errors. The first observation is that all experimental results fall within these whiskers, with a lower dispersion, which is at least in part due to their limited number (five measurements per each case). A little positive bias is also observable, especially in the two cases of leak position equal to 7.5 m and $\sigma_{s}=[5\;ns,\;7\;ns]$. This bias can be attributed to the reduced temporal resolution associated with wider stimulus pulses. In these conditions, the reflection associated with the water region spreads out more (as can be seen in Figure 6), and this broadening is not perfectly captured by the model used in the inversion procedure, resulting in a small bias in the estimated position.

Table 4 summarizes the mean and standard deviation of localization errors obtained from the experimental measurements, grouped by stimulus pulse duration and leak position (for each position, the lowest mean and standard deviation are highlighted in bold). Compared to the simulation results in Table 3, experimental errors—as shown by Figure 13—exhibit lower standard deviation and slightly higher bias for some configurations, though never exceeding 3.9 cm. The maximum absolute error over all measurements is 4.7 cm.

Summing up:

- According to hundreds of simulations, leak localization errors lie, in 95% of the cases, within about ±12 cm when considering all simulated cases, with a negligible bias (4 mm);
- The 45 experimental results are all contained within these bounds and exhibit comparable bias and dispersion, confirming the quantitative agreement between simulations and measurements;
- The mean localization error is consistently small in both simulations and experiments, remaining within ±1.7 cm in simulations and ±3.9 cm in experiments across all tested conditions;
- The standard deviation of the error is larger in simulations (approximately 3.9–6.8 cm) than in experiments (approximately 0.25–1.8 cm), indicating that the simulation-based uncertainty estimates are slightly conservative;
- The differences between simulations and experiments (e.g., in standard deviation and bias of the estimates) are of no practical relevance, and can be explained by the simplifications and approximations introduced by the simulation model, and also by the limited number of laboratory experiments.

### 4.3. Comparison with Time-of-Flight-Based Localization

**Figure 14:**
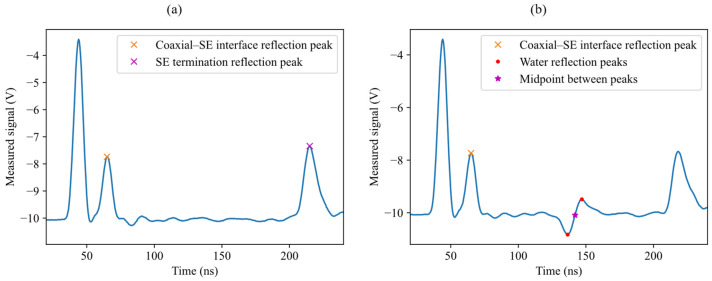
Time-of-flight-based localization procedure. (**a**) Identification of reference reflections used for propagation velocity estimation, namely the coaxial–SE interface reflection and the reflection at the end of the line. (**b**) Identification of the leak reflection. The time of flight was computed as the difference between the midpoint between the negative and positive peaks of the leak reflection and the interface reflection.

For comparison purposes, a conventional time-of-flight-based localization method was also applied to the same experimental data. In this approach, the propagation velocity was estimated from measurements acquired in dry conditions, by evaluating the time difference between the reflection at the coaxial cable–SE interface and the reflection at the end of the line, as illustrated in Figure 14a. The leak position was then determined by identifying the reflection associated with the water region. The corresponding time of flight was computed as the difference between the midpoint between the negative and positive peaks of the leak reflection and the time instant of the coaxial cable–SE interface reflection (Figure 14b). This time interval was then converted into spatial coordinates using the estimated propagation velocity.

The obtained localization errors show a significantly larger dispersion compared to the proposed inversion-based method. In particular, the mean error is 10.1 cm, with a standard deviation of 12.8 cm, and a 95% interval approximately spanning from −13.7 cm to 25.5 cm. By contrast, the proposed method yields substantially smaller experimental errors, with a maximum absolute error of 4.7 cm over all measurements.

### 4.4. Limitations and Applicability of the Proposed Framework

The proposed approach is based on a gray-box model that can represent spatial variations in the dielectric properties along the sensing element, and can therefore be extended to conditions involving gradual moisture changes, such as partial saturation of the surrounding medium. In such cases, the reduced dielectric contrast leads to weaker reflections and, consequently, to increased uncertainty in the localization result. Similarly, the method can be applied to longer sensing elements, although increased attenuation, dispersion, and reduced signal-to-noise ratio may affect performance.

It is important to note that the main contribution of this work lies not only in the refined inversion method, but also in the proposed framework for uncertainty evaluation. This framework is general and can be applied to different operating conditions and setups, as well as to TDR-based localization methods other than the one proposed here. In particular, for scenarios such as partial saturation, extended sensing lengths, or more complex environmental conditions, the same simulation-based approach can be used to perform dedicated Monte Carlo analyses and quantify the corresponding uncertainty, provided that the gray-box model is appropriately adapted to reproduce the reflectograms under the conditions of interest.

These aspects are not explicitly explored in the present study and therefore represent a limitation with respect to direct applicability to real field conditions. In particular, the sensing element is relatively short and is laid on the floor rather than being buried in soil, and the water regions are introduced in a controlled manner. While this configuration is not fully representative of practical installations, it allows the acquisition of a large number of measurements with accurately known leak positions, which is essential for a reliable and statistically meaningful characterization of the localization uncertainty. At the same time, these controlled experimental conditions enable a direct validation of the proposed uncertainty evaluation framework.

## 5. Conclusions

This study focuses on the uncertainty analysis of a water leak localization method based on a distributed sensing element and TDR inversion. Building upon a previously introduced inversion framework, the method’s implementation was refined and its performance thoroughly assessed through extensive simulations and laboratory experiments.

Both the localization and uncertainty evaluation rely on a realistic “gray-box” circuital model of the measurement setup, capable of reproducing key features of real reflectograms while remaining simple enough for reliable parameter identification. Disturbance phenomena are represented in a simplified yet effective way, via random capacitance variations.

Hundreds of simulated measurements with different leak positions, stimulus pulse durations, and other influencing factors, yielded 95% confidence intervals for the localization error, together with bias and standard deviation estimates. For the examined 15 m sensing element, with 60 cm leaks in three positions and three different pulse durations, the 95% confidence interval was about ± 12 cm, with a bias < 1.7 cm and a standard deviation < 7 cm.

A total of 45 experimental measurements confirmed the uncertainty evaluation: all errors fell within the simulation-based bounds, with lower dispersion, a slightly higher bias, and a maximum absolute error of 4.7 cm. The agreement between simulations and experiments demonstrates both the accuracy of the uncertainty evaluation, and the robustness of the localization method in practical conditions.

The demonstrated accuracy and consistency suggest that the proposed approach can serve as a robust tool for TDR-based leak localization, supporting both practical leak detection tasks and formal uncertainty evaluation, and offering a framework readily adaptable to other distributed sensing applications.

## Figures and Tables

**Table 1:** Result of the measurement in Figure 5, in terms of primary parameters of the coaxial cables and of the SE.

Parameter and Measurement Unit	Value
$R_{0,c}\;\left(\frac{\Omega }{m}\right)$	$8.1961\times 10^{-2}$
$R_{1,c}\;\left(\frac{\Omega }{m\;\sqrt{Hz}}\right)$	$2.5294\times 10^{-4}$
$L_{0,c}\;\left(\frac{H}{m}\right)$	$2.6636\times 10^{-7}$
$L_{1,c}\;\left(\frac{H}{m}\right)$	$4.3044\times 10^{-8}$
$f_{0,c}\;(Hz)$	$3.8483\times 10^{3}$
$G_{0,c}\;\left(\frac{S}{m\;Hz}\right)$	$3.8164\times 10^{-12}$
$C_{0,c}\left(\frac{F}{m}\right)$	$9.6806\times 10^{-11}$
$R_{0,SE}\;\left(\frac{\Omega }{m}\right)$	$1.5777\times 10^{-3}$
$R_{1,SE}\;\left(\frac{\Omega }{m\;\sqrt{Hz}}\right)$	$4.4029\times 10^{-4}$
$L_{0,SE}\;\left(\frac{H}{m}\right)$	$5.3402\times 10^{-7}$
$L_{1,SE}\;\left(\frac{H}{m}\right)$	$9.5390\times 10^{-7}$
$f_{0,SE}\;(Hz)$	$2.0173\times 10^{4}$
$G_{0,SE}\;\left(\frac{S}{m\;Hz}\right)$	$6.7611\times 10^{-12}$
$C_{0,SE}\left(\frac{F}{m}\right)$	$4.4958\times 10^{-11}$

**Table 2:** Summary of nominal parameters in experimental tests.

Number of physical SEs	5
Length of the SEs	15 m
Water region center locations	3.5 m, 7.5 m, 11.5 m
Water region width	60 cm
Standard deviations of stimulus Gaussian pulses	3.1 ns, 5 ns, 7 ns
Total number of experimental measurements	45

**Table 3:** Mean and standard deviation of water leak center estimation errors in simulations.

$\mathrm{Leak}\;\mathrm{Center}\;z_{c}$	3.5 m			7.5 m			11.5 m		
Trans. Pulse Std $\sigma_{s}$	3.1 ns	5 ns	7 ns	3.1 ns	5 ns	7 ns	3.1 ns	5 ns	7 ns
**Mean error** **(m)**	−0.0163	−0.0165	**−0.0134**	**−0.0033**	−0.0037	−0.0066	**−0.0006**	0.0075	0.0145
**Standard deviation of error** **(m)**	**0.0425**	0.0525	0.0583	**0.0502**	0.0579	0.0678	**0.0391**	0.0457	0.0609

**Table 4:** Mean and standard deviation of water leak center estimation errors in experimental tests.

$\mathrm{Leak}\;\mathrm{Center}\;z_{c}$	3.5 m			7.5 m			11.5 m		
Trans. Pulse Std $\sigma_{s}$	3.1 ns	5 ns	7 ns	3.1 ns	5 ns	7 ns	3.1 ns	5 ns	7 ns
**Mean error** **(m)**	**0.0018**	0.0099	0.0104	**−0.0059**	0.0195	0.0386	**0.0049**	0.0164	0.0152
**Standard deviation of error** **(m)**	**0.0091**	0.0165	0.0174	**0.0025**	0.0105	0.0062	0.0126	0.0079	**0.0070**

## Data Availability

The original contributions presented in this study are included in the article. Further inquiries can be directed to the corresponding author.
